# Cancer and non-cancer health effects from food contaminant exposures for children and adults in California: a risk assessment

**DOI:** 10.1186/1476-069X-11-83

**Published:** 2012-11-09

**Authors:** Rainbow Vogt, Deborah Bennett, Diana Cassady, Joshua Frost, Beate Ritz, Irva Hertz-Picciotto

**Affiliations:** 1 UC Davis, Department of Public Health Sciences, 1 Shields Avenue, Davis, CA, 95616, USA; 2 UC Los Angeles, Department of Epidemiology, 100 Stein Plaza Driveway, Los Angeles, CA, 90095, USA

**Keywords:** Dietary toxic exposure prevention, Nutritional toxicology, Organic food, Cancer risk, Chemical contaminants in food

## Abstract

**Background:**

In the absence of current cumulative dietary exposure assessments, this analysis was conducted to estimate exposure to multiple dietary contaminants for children, who are more vulnerable to toxic exposure than adults.

**Methods:**

We estimated exposure to multiple food contaminants based on dietary data from preschool-age children (2–4 years, n=207), school-age children (5–7 years, n=157), parents of young children (n=446), and older adults (n=149). We compared exposure estimates for eleven toxic compounds (acrylamide, arsenic, lead, mercury, chlorpyrifos, permethrin, endosulfan, dieldrin, chlordane, DDE, and dioxin) based on self-reported food frequency data by age group. To determine if cancer and non-cancer benchmark levels were exceeded, chemical levels in food were derived from publicly available databases including the Total Diet Study.

**Results:**

Cancer benchmark levels were exceeded by all children (100%) for arsenic, dieldrin, DDE, and dioxins. Non-cancer benchmarks were exceeded by >95% of preschool-age children for acrylamide and by 10% of preschool-age children for mercury. Preschool-age children had significantly higher estimated intakes of 6 of 11 compounds compared to school-age children (p<0.0001 to p=0.02). Based on self-reported dietary data, the greatest exposure to pesticides from foods included in this analysis were tomatoes, peaches, apples, peppers, grapes, lettuce, broccoli, strawberries, spinach, dairy, pears, green beans, and celery.

**Conclusions:**

Dietary strategies to reduce exposure to toxic compounds for which cancer and non-cancer benchmarks are exceeded by children vary by compound. These strategies include consuming organically produced dairy and selected fruits and vegetables to reduce pesticide intake, consuming less animal foods (meat, dairy, and fish) to reduce intake of persistent organic pollutants and metals, and consuming lower quantities of chips, cereal, crackers, and other processed carbohydrate foods to reduce acrylamide intake.

## Background

Food may be the primary route of exposure to contaminants from multiple chemical classes such as metals (mercury, lead, arsenic), persistent organic pollutants (POPs) (dioxin, DDT, dieldrin, chlordane), and pesticides (chlorpyrifos, permethrin, endosulfan). Food toxicology assesses exposure to contaminants from typical diets and their related health outcomes. Though food-borne toxic contaminants are a concern for all ages, they are of greatest concern for children, who are disproportionately impacted because they are still developing and have greater intake of food and fluids relative to their bodyweight. Pediatric problems that have been linked to preventable environmental toxin exposures include cancer, asthma, lead poisoning, neurobehavioral disorders, learning and developmental disabilities, and birth defects [1,2].

Dietary practices influence exposure to pesticides, metals, persistent organic pollutants, and industrial pollutants through consumption patterns, food packaging, and preparation methods. A diet high in fish and animal products, for example, results in greater exposure to persistent organic compounds and metals than does a plant-based diet because these compounds bioaccumulate up the food chain. Besides varying by types of food eaten, exposure from our diet depends on consumption frequency and amount consumed, as well as growing conditions of crops such as pesticide use, soil characteristics, and water source. The way in which food is cooked, processed, and packaged may introduce chemicals such as bisphenol A, phthalates, and acrylamide that are not present in the raw food [3-5]. Because bisphenol A has been detected in baby food, this compound has been banned in the production of plastic baby bottles in Canada, the European Union, Denmark, and a growing number of U.S. states.

The implementation of the Food Quality Protection Act of 1996 (FQPA) has resulted in significant enhancements in public use databases reporting on levels of toxic compounds in food [6]. In addition to setting tolerance levels for registered chemicals, the FQPA requires the U.S. Environmental Protection Agency to consider aggregate risk from exposure to a pesticide through multiple sources and cumulative risk from exposure to pesticides that have common mechanisms of toxicity. To date, many studies of dietary exposure to harmful substances focus on a single chemical or compound, for example chlordane or mercury [7,8]. Still needed are exposure assessments that comprehensively consider the broad array of food contaminants found in a typical diet.

Aggregate risk exposure comprehensively considers the multiple toxins to which people are exposed on a daily basis throughout the life span, including during sensitive developmental periods such as pregnancy and childhood. In a recent analysis of pregnant women in the U.S. (n=268), certain polychlorinated biphenyls, organochlorine pesticides, perfluorinated chemicals, phenols, polybrominated diphenyl ethers, phthalates, polycyclic aromatic hydrocarbons, and perchlorate were detected in 99-100% of pregnant women [9]. A number of pesticides and industrial or household compounds from various chemical classes are categorized as endocrine disrupting contaminants (EDCs) because they exhibit high potency in very small amounts and are capable of disrupting reproductive, developmental, and other hormonally mediated physiological functions [10]. Many EDCs are also categorized as POPs— including compounds such as banned pesticides and unwanted byproducts of industrial processes and waste incineration that accumulate and persist in the environment and the human body [11].

Studies that assess multiple exposures improve our understanding of how different compounds may act synergistically to cause greater damage than would be incurred by a single exposure. In one study examining the impact of multiple chemical exposures on functional abilities, researchers found that Mexican children (n=50) who had been exposed to multiple pesticides in pregnancy and childhood had less stamina, poorer eye-hand coordination, poorer memory and were less skilled in drawing figures compared to their less exposed counter-parts [12].

Studies assessing multiple dietary contaminants and their exposures in children are relatively uncommon, and we found no previous reports covering the specific group of contaminants included in the present analysis (acrylamide, arsenic, lead, mercury, chlorpyrifos, permethrin, endosulfan, dieldrin, chlordane, DDE, and PCCD/PCDFs). Three previous studies present child-specific dietary and contaminant data [13-15] while others present adult data only [16-19]. Previous articles present data on food contaminant levels both estimated [13-16,18-21] and measured [17,22,23]. Here, we estimate, for three different age groups, exposures for which food serves as a main source. Based on parent- or self-reported food intake for children, adults with young children, and older adults, we derived exposure estimates for eleven toxic compounds. We also estimated which foods contributed most to these exposures based on the frequency and quantities eaten, and identified compounds that are estimated to exceed non-cancer and cancer benchmarks and therefore would pose health risks of particular concern.

## Methods

The primary dataset for this analysis was provided by the Study of Use of Products and Exposure-Related Behavior (SUPERB). SUPERB was funded by the U.S. Environmental Protection Agency (US EPA) for the purpose of examining behaviors in three domains that influence human exposure to environmental chemicals: food consumption, personal care/household care product use, and temporal-spatial activity; the complete methodology for this study and a description of participant demographics are reported separately [24]. Participants were recruited from 21 counties in California and from two types of households: (1) households with a child under age 6 and a parent, from the northern California region and (2) households with an adult ≥55 years of age, from the central California region. Results are presented by age group: preschool-age children (ages 2–4, referred to hereafter as preschoolers), school-age children (ages 5–7, referred to hereafter as school-age children); adults with young children (referred to hereafter as parents); and older adults (referred to hereafter as older adults). The study was approved by both the UC Davis and UCLA Institutional Review Boards and the State of California Institutional Review Board (see [24], for a full description of the methodology). This report utilizes the food frequency data to quantify intake of selected dietary toxic chemical contaminants.

### Determining food consumption rates

Food consumption data used for this analysis were collected during the SUPERB first-year telephone-administered survey (in 2007), conducted in English and Spanish. Trained interviewers administered an abbreviated instrument based on a standard food frequency questionnaire (FFQ); we used an abbreviated version because our interest was not in total nutritional intake but rather sources of contaminants. This version has not been validated specifically, although the original survey has been validated [25]. We asked about the frequencies and amounts of foods typically eaten in the last year; adults answered for themselves and their children. We reduced 126 food items to 44 to cover key foods and food groups associated with a higher risk of exposure to specified toxins and classes of toxic compounds selected a priori.

Contaminants were selected to represent different chemical classes, food groups, and health effects: metals (arsenic, lead, mercury), pesticides (chlorpyrifos, permethrin, endosulfan), persistent organic pollutants (POPs) (dioxin, DDT, dieldrin, chlordane), and processing additives/byproducts (acrylamide, hormones, antibiotics). For acrylamide, we included foods for which high levels were reported, based on an analysis that combined Food and Drug Administration (FDA) data on acrylamide levels in U.S. foods with survey data generated by the U.S. Department of Agriculture and other organizations on food consumption rates [26]. For pesticides, we focused on fruits and vegetables shown to have the highest residues in analyses conducted by the Environmental Working Group (EWG), which ranks pesticide contamination for popular fruits and vegetables based on an analysis of 87,000 tests of these foods, conducted between 2000 and 2007 by the U.S. Department of Agriculture and the Food and Drug Administration [27]. The produce listed in the EWG’s analysis was chosen based on an analysis of USDA food consumption data from the Continuing Survey of Food Intakes by Individuals (CSFII) 1994–1996 [28]. For pesticide and other compounds that bioaccumulate or are persistent, we collected animal-based items, i.e., dairy, eggs, meat, and fish sources shown to be high in these contaminants [29]; for mercury, the primary source is fish. Because public use datasets with estimated exposure levels for antibiotics and hormones in food were not available from the FDA, we did not pursue these two classes of compounds.

### Determining contaminant concentrations in food

From the reported consumption of targeted food items, we estimated levels of intake for the remaining chemicals of concern using existing databases and reports (see Table 1). The Total Diet Study (TDS) served as the basis for these calculations [29-31]. Food intake of five pesticides, namely, chlorpyrifos, permethrin, endosulfan, dieldrin and 1,1-dichloro-2,2-bis(p-chlorophenyl)ethylene (DDE, the major and persistent DDT metabolite), the latter two being POPs that have been banned, were estimated from the mean TDS values for each food item [29]. To estimate intake of polychlorinated dibenzo-p-dioxins (PCDDs) and polychlorinated dibenzofurans (PCDFs), a separate TDS dataset was used containing mean PCDD/F concentrations weighted by toxic equivalents (TEQ) [31]; if the reported sample was below the limit of detection, we set the value equal to the limit of detection divided by the square root of two. We quantified acrylamide based on a compilation by the California Environmental Protection Agency [26] that used mean levels from the Total Diet Study of Acrylamide in Food. Intakes of arsenic and lead were calculated using mean levels reported in the TDS [29], while that of mercury was estimated from US Health and Human Services and USEPA data (mean values used), made available through the US FDA Center for Food Safety and Applied Nutrition program [30]. These data are estimates of total mercury including methylmercury, which is directly proportional to total mercury concentrations and was shown to compose 83% of total mercury in fish muscle concentrations [32].

**Table 1:** Sources of data for food contaminants

**Contaminant**	**Source of Data for Food Contaminant Levels**	**Source of Non**-**cancer References Dosages**	**Source of Cancer Potency Factors**
Arsenic, Dieldrin, Chlordane, DDT	Mean values reported from the Total Diet Study (USFDA, 2003) [29]	IRIS Database (USEPA, 2010) [33]	EPA (USEPA, 2010) [33]
Lead, Chlorpyrifos, Permethrin, Endosulfan	Mean values reported from the Total Diet Study (USFDA, 2003) [29]	IRIS Database (USEPA, 2010) [33]	Not available
Mercury	Mean values from USHHS and USEPA data (USFDA, 2006) [30]	IRIS Database (USEPA, 2010) [33]	Not available
PCDD/Fs	Mean concentrations reported from the TDS weighted by toxic equivalent (USFDA, 2004a) [31]	Provisional tolerable monthly intake established by the World Health Organization (JECFA, 2001) [34]	EPA Dioxin Reassessment for 2,3,7,8-Tetrachlorodibenzo-*p*-Dioxin (TCDD) (USEPA, 2000) [35]
Acrylamide	USDA Total Diet Study of Acrylamide (CEPA, 2005) [26]	IRIS Database (USEPA, 2010) [33]	Not available

### Estimating daily intake

Person-specific estimates for intake of each compound of interest were determined based on responses of SUPERB Study participants to the FFQ for the 44 items, in combination with estimated contaminant levels by food item for each of the chemicals or compounds of interest listed in the existing databases. We included fluids such as juices, but not water or water-based drinks such as coffee or tea. Dietary data were collected from participants by asking the frequency of consumption of food items and the usual serving sizes, which were converted into grams. From these data, food intake was calculated as the average g/day; then, similar to other studies, we adjusted for self-reported body weight of participants, obtaining g food/kg body weight per day [14,36]. Thus, average daily contaminant intake was calculated for each individual as follows:

(1) $$\begin{array}{ll} \sum_{i} & \left(\mu g\;\text{contaminant}/\text{kg}\;\text{bw}\;\text{per}\;\text{day}\right)\\ & =\sum_{i}\left(g\;{\text{food}}_{i}/\text{kg}\;\text{bw}\;\;\text{per}\;\text{day}\right)\\ & \;\times \left(\mu g\;\text{contaminant}/g\;{\text{food}}_{i}\right) \end{array}$$

where i= the i^th^ food item containing the contaminant of interest, and bw stands for body weight. These were then averaged across individuals within each age group. In the case that data were available for multiple isomers of a contaminant, contaminant levels were summed and assigned to the given food. Some foods with multiple ‘types’ were asked as a group (e.g. tortilla and potato chips) in which case the food selected for estimating the contaminant level was that consumed in the greatest amount, according to the national TDS data (e.g. for chip consumption, tortilla chips were used to estimate contaminant levels because they are consumed in higher amounts than potato chips). To gauge relative accuracy of self-reported dietary intake, we compared reported quantities of foods consumed by SUPERB participants with national TDS data. We also calculated t-tests to assess differences in estimated toxin intake between age groups.

### Determining dose–response and risk

Finally, we used non-cancer benchmarks to calculate “hazard ratios” and cancer benchmarks to calculate risk ratios. We defined the “hazard ratios” as the estimated intake for a chemical divided by the non-cancer benchmark, based on Oral Reference Doses (RfDs) available from the U.S. EPA's Integrated Risk Information System (IRIS) database. RfDs are defined as threshold levels for daily intake that are likely to result in no appreciable risk of a deleterious effect during a lifetime [33]. (Above this threshold, a “hazard” exists, hence the “hazard ratio.”) Non-cancer benchmarks from the EPA were used for the following compounds (and their isomers): acrylamide, arsenic, lead, methylmercury, chlorpyrifos, dieldrin, endosulfan (I- and II-), permethrin (cis- and trans-), p,p'-dichlorodiphenyldichloroethylene (DDE), and chlordane (cis- and trans-). The only exception was the benchmark level for PCDD/Fs, for which a provisional tolerable monthly intake established by the World Health Organization was used as the non-cancer benchmark since the RfD is unavailable through the IRIS database [34].

For carcinogens, we calculated risk ratios by dividing chemical intakes by cancer benchmarks established by the EPA. The cancer benchmarks are based on cancer potency factors (CPFs), which are used to estimate contaminant intake values that would be expected to cause no more than one excess cancer in a million (10^-6^) persons exposed over a 70-year life span. CPFs were available through IRIS for arsenic, dieldrin, DDE, chlordane and from the EPA Dioxin Reassessment for 2,3,7,8-Tetrachlorodibenzo-p-Dioxin (TCDD), the most toxic of the PCDD/F congeners, which can be applied to summed TEQ values [35]. We compared non-cancer and cancer benchmark levels with our population intake distribution to assess potential health impacts. We also report descriptive statistics of daily mean intake per kg bodyweight (mean, standard deviation, inter-quartile range, 10^th^, 50^th^ and 90^th^ percentiles) and the percentage of participants above the non-cancer and cancer benchmarks for all age groups. We determined the top five foods contributing to exposure for a given chemical based on daily intake calculated for each contaminant. The range for the percent contribution of each of the 5 main food items to the total toxic load of the given contaminant was also calculated, i.e., we report the lowest to highest percentage contribution across all individuals who consumed that food.

### Missing data, outliers, and assumptions

Among children, ages 2–7, (n=365), 24 were missing weight. Among adults ages 18+ (n=599), 52 were missing weight. We used multiple imputation with the Markov Chain Monte Carlo method (SAS/STAT 9.2, PROC MI) to impute the missing weights and heights. Separate imputation models were developed for each age group. Variables for each imputation model were identified by calculating correlations of weight with dietary and demographic variables for each age group (see Additional file 1: Table S1 for variables). For t-tests and Pearson correlations, variables were included in the corresponding imputation model if the probability of a random difference was below 20% and cell counts were five or greater. Imputation was carried out five times, for which the acceptable range was calculated from known values (limits: 1st percentile minus (0.5)*(SD), 99th percentile plus (0.5)*(SD)). The average of the five imputed values was used as a point estimate of the missing weight and height values. For analysis of variance, all five sets of imputed values were used (SAS/STAT 9.2, PROC MIANALYZE).

In four food categories (red meat, poultry, fish, and dairy products), we collected frequency of use data but not the usual food serving amount. We imputed serving size based on participant responses for the other foods (at least three other foods with serving size information) whenever possible. There remained 13 participants with incomplete data on those four food items who were excluded from the relevant analyses. Similarly, the “usual serving size” response (i.e., the smaller, middle, or larger serving size selected by the participant) was missing for 72 food items in interviews with 55 adults, and 68 items for 51 children. For all of these participants (except one adult), we had at least three measurements of serving size from other foods, and used these values to impute the missing value. The serving size estimates were not validated other than in the original validation of the questionnaire [25].

For food frequency outliers that seemed too large to be accurate (e.g., ≥70 servings of melon per week), if the weekly frequency was greater than 21 it was divided by seven, under the assumption that the interviewer accidentally selected the adjacent unit of time, entering ‘per day’ instead of ‘per week’ (17 instances among 17 adults; 20 instances among 19 children). Exceptions were made for higher consumption food categories including juice and dairy products: for these items, we used a cut point of 50 per week, above which the frequency was divided by seven (19 instances among 19 adults; none among children). We collected frequency of fruits eaten in and out of season (for six fruits) but we only collected one serving size per fruit. We assumed that fruit serving size was independent of season.

## Results

Table 2 presents the percent of preschoolers who exceed benchmark intakes (noncancer benchmarks in the 3^rd^ to the last column, cancer benchmarks in the final column) adjusted for body weight (data for school-age children, parents, and older adults are included in Additional file 2: Table S2). Children’s estimated intakes most commonly exceeded the non-cancer benchmark for acrylamide (≥95% of children), lead (100% of children) and DDE (100% of children); the reference values for both lead and DDE are zero. Other non-cancer benchmarks that were exceeded included mercury (10% of preschoolers and 6% of school-age children), and PCCD/PCDFs (2% of preschoolers). Preschool children’s intake exceeded the cancer benchmarks for arsenic, dieldrin, DDE, and PCCD/PCDFs (100%), and for chlordane (6.8% of preschoolers and 3.8% of school-age children). Preschoolers were significantly more likely to have higher intakes, for their body weight, of acrylamide (p=0.0003), lead (p=0.002), chlordane (p=0.0003), dieldrin (p=0.02), DDE (p<0.0001), and PCCD/PCDFs (p=0.0001) as compared with school-age children. As compared with parents, preschoolers were significantly more likely to be exposed to acrylamide, arsenic, lead, mercury, chlorpyrifos, endosulfan, chlordane, dieldrin, DDE, and PCCD/PCDFs (all at p<0.0001), which is partly explained by our parameterization of intake on a per body weight basis. These higher exposures emphasize the general concern about greater intake, on a weight-for-weight basis, in very young children. The same pattern was observed comparing school-age children with parents. Parents, as compared with adults 55 or older, had significantly higher intakes of acrylamide, chlordane, DDE, and PCCD/PCDFs (all at p<0.0001) (data not shown).

**Table 2:** **Intake of food contaminants in preschool**-**aged children** (**mg/kg**/**day**)

Preschool-aged Children (2–4) (n=207)*										
**Toxin**	**Descriptive exposure statistics**						**Benchmarks dosages**			
	**N exposed based on dietary data**	**Mean daily intake (mg/kg bodyweight/ day)**^†^	**SD of daily intake (mg/kg bodyweight/ day)**	**10**^**th**^**percentile of daily intake (mg/kg bodyweight/ day)**	**median of daily intake (mg/kg bodyweight/day)**	**90th percentile of daily intake (mg/kg bodyweight/day)**	**Reference Dosage (RfD) (mg/kg bodyweight/day)**	**% participants > RfD**	**Cancer Benchmark (CB) (mg/kg bodyweight/ day)**^†^^†^	**% participants > Cancer Benchmark**
**Acrylamide**	207	**1**.**18E**-**03**	9.48E-04	3.17E-04	9.22E-04	2.51E-03	0.0002	97.10%	-	-
**Metals**									-	
Arsenic	207	**1**.**98E**-**04**	2.37E-04	3.84E-05	1.02E-04	4.03E-04	0.0003	18.36%	6.67E-07	100.0%
Lead^‡^	207	**1**.**36E**-**04**	6.92E-05	5.90E-05	1.18E-04	2.35E-04	0.000^‡^	100.00%	-	-
Methylmercury	147	3.17E-05	5.68E-05	1.40E-06	2.53E-05	1.02E-04	0.0001	10.20%	-	-
**Current use pesticides**										
Chlorpyrifos	207	7.45E-05	6.56E-05	1.60E-05	5.59E-05	1.51E-04	0.003	0.00%	-	-
Permethrin	207	1.29E-04	1.60E-04	3.06E-06	7.35E-05	3.32E-04	0.05	0.00%	-	-
Endosulfan	207	4.01E-05	3.31E-05	1.07E-05	3.16E-05	8.95E-05	0.006	0.00%	-	-
**Persistent organic pollutants**										
Chlordane	207	**1**.**54E**-**06**	8.45E-07	4.84E-07	1.46E-06	2.63E-06	0.0005	0.00%	2.86E-06	6.76%
Dieldrin	207	**4**.**17E**-**06**	3.03E-06	1.52E-06	3.36E-06	7.82E-06	0.00005	0.00%	6.25E-08	100.00%
DDE^‡^	207	**3**.**39E**-**05**	1.78E-05	1.36E-05	3.12E-05	5.51E-05	0.000^‡^	100.00%	2.94E-06	100.00%
PCDD/Fs	207	**1**.**01E**-**09**	4.78E-10	4.74E-10	9.34E-10	1.58E-09	2.30E-09	2.42%	1.00E-12	100.00%

^†^ Boldface values represent whole-population average estimated exposures that exceed the non-cancer or cancer benchmarks.

^††^ Cancer benchmarks equal 10^-6^ divided by the cancer slope factor and represents the exposure concentration at which lifetime cancer risk is one in one million.

The source is the EPA IRIS except for PCDD/Fs, for which the cancer potency factor for TCDD was used from the EPA dioxin reassessment (2003) [34].

^‡^ The EPA has concluded that setting RfDs for lead is inappropriate because effects occur at levels so low as to be essentially without a threshold. The EPA has also not set a reference dosage for DDE thus both are presented as having a RfD of zero.

**Figure 1:**
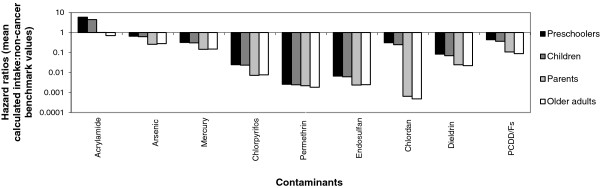
Hazard ratios using Reference Dosages for contaminants.

**Figure 2:**
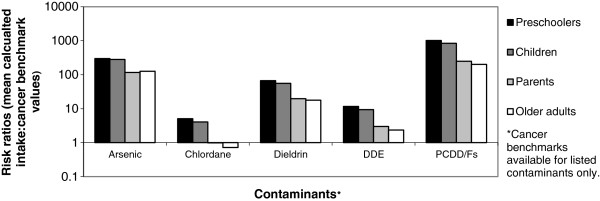
Hazard ratios of Cancer Benchmarks for contaminants.

Figures 1 and 2 show the non-cancer benchmark hazard ratios and cancer benchmark risk ratios, respectively, which compare population mean intakes to the non-cancer and cancer benchmark levels. These figures indicate the magnitude of exposure: values that are >1 indicate the –fold factor by which the estimated mean intake exceeds the benchmark levels. These benchmark hazard or risk ratios are shown for each of the four age groups. For acrylamide, non-cancer benchmark hazard ratios (Figure 1) exceeded 1 for all groups except for older adults. Thus, the estimated mean intake exceeds the acrylamide benchmark increasingly as age decreases, rising to 5.9 for preschoolers. The RfDs for lead and DDE have not been set (see final footnote to Table 2); we used zero for both and hence any intake, including the mean, exceeds those non-cancer benchmarks. Mean intake for other chemicals did not exceed the noncancer benchmarks.

The cancer benchmark risk ratios are shown in Figure 2 and indicate that the benchmarks were exceeded by all five contaminants for which there was a cancer benchmark (arsenic, dieldrin, DDE, PCCD/PCDFs and chlordane), in school-aged and preschool children. This was also true for adults with the exception of chlordane, where mean intake was slightly below the benchmark. The ratio of intake level to cancer benchmark ranged from 116–297 for arsenic, 4–5 for chlordane (among children), 18–67 for dieldrin, 2–12 for DDE, and 202–1010 for PCDD/Fs.

The top individual foods contributing to intake of a given contaminant were similar across age groups (Table 3 shows data for preschoolers; the other age groups are presented in Additional file 3: Table S3). Meat, dairy, cucumber and potatoes were top contributors, for all age groups, to POPs (chlordane, dieldrin, DDE, and PCDD/Fs. Other major contributors to POPs were poultry, freshwater fish, cantaloupe, mushrooms, as well as spinach (adults only) and pizza (children only). Dairy was also a main contributor to lead exposure among all age groups and to chlorpyrifos among children. Produce items such as tomatoes, peaches, apples, peppers, grapes, lettuce, broccoli, strawberries, spinach, pears, green beans, and celery were the top contributors to current use pesticides (chlorpyrifos, permethrin, and endosulfan). Poultry, salmon, tuna, and mushrooms were top contributors to arsenic in all age groups. Fried potatoes, chips, cereal, and crackers were top contributors to acrylamide in all age groups. Additional foods that have high levels of contamination that were not captured by the top five ranks in Table 3 (due to lower reported consumption) include popcorn and pretzels for acrylamide, spinach for lead, fish (fresh and saltwater) for dieldrin, and lettuce and celery for DDE.

**Table 3:** **Top five food sources for contaminants in preschool**-**aged children**^**a**^

**Toxins (RfD) μg/kg/day**	**Preschool-aged Children 2–4 yrs old (n=207)**				
	Highest	2nd	3rd	4th	5^th^
**Acrylamide (0.2 μg/kg/day)**	crackers	fried potatoes^b^	cereal	graham crackers	chips
Mean μg/kg/day	0.434607	0.257568	0.188175	0.574920	0.323679
n	125	118	100	94	71
% range	3.9-82.4%	8.9-76.7%	2.2-70.4%	6.2-84.1%	4.2-96.6%
**Metals**	Highest	2nd	3rd	4th	5^th^
** Arsenic** (**0**.**3μg**/**kg**/**day**)	poultry^b^	cereal	salmon	tuna	mushrooms
Mean μg/kg/day	0.022756	0.014547	0.141837	0.183135	0.052914
n	148	81	77	70	61
% range	0.6-74.7%	2.3-47.9%	2.3-97.6%	10.3-96.4%	0.6-81.9%
** Lead** (**0**.**0μg**/**kg**/**day**)*	dairy^b^	apple juice	grapes	cookies	sweet potatoes^c^
Mean μg/kg/day	0.032679	0.043302	0.017289	0.015441	0.039843
n	184	112	58	42	36
% range	7.1-83.8%	4.8-78.3%	3.2-35.5%	5.2-40.0%	7.5-63.1%
** Methylmercury** (**0**.**1μg**/**kg**/**day**)	seafood^b^	tuna	freshwater fish^b^	n/a	n/a
Mean μg/kg/day	0.030454	0.033099	0.015439	n/a	n/a
n	91	72	69	n/a	n/a
% range	1.6-100%	5.7-100%	0.7-100%	n/a	n/a
**Pesticides**	Highest	2nd	3rd	4th	5th
** Chlorpyrifos** (**3**.**0µg**/**kg**/**day**)	grapes	apples	peaches	dairy^b^	tomatoes
Mean μg/kg/day	0.027609	0.017583	0.019758	0.002052	0.006011
n	179	174	127	49	28
% range	3.9-86.8%	3.9-91%	2.4-69.9%	1.2-60.8%	2.7-48.9%
** Permethrin** (**50**.**0μg**/**kg**/**day**)	lettuce	spinach	broccoli^b^	tomatoes	peaches
Mean μg/kg/day	0.072562	0.138982	0.005281	0.014693	0.003968
n	108	103	102	86	59
% range	4.9-98.5%	3.4-99.4%	0.5-99%	1.2-98.7%	0.2-98.9%
** Endosulfan** (**6**.**0μg**/**kg**/**day**)	apples	peaches	strawberries	tomatoes	pears
Mean μg/kg/day	0.008055	0.009462	0.006409	0.009509	0.006667
n	129	99	94	75	48
% range	1.2-79.4%	7.6-77.5%	4.8-62.1%	8.8-85.8%	7.2-63.3%
**POPs**	Highest	2nd	3rd	4th	5th
** Chlordane** (**0**.**5μg**/**kg**/**day**)	dairy^b^	cucumber	meat^b^	popcorn	potatoes
Mean μg/kg/day	0.012221	0.003734	0.000745	0.000844	0.000985
n	200	96	94	65	55
% range	1.0-99.8%	1.4-96.3%	1.0-55.0%	0.8-20.1%	0.3-44.2%
** Dieldrin** (**0**.**05μg**/**kg**/**day**)	dairy^b^	meat^b^	cucumber	cantaloupe	pizza
Mean μg/kg/day	0.001563	0.000606	0.002496	0.000547	0.000172
n	195	108	101	63	40
% range	5.7-96.0%	1.4-76.4%	5.2-93.6%	3.4-61.7%	1.1-56.6%
** DDE** (**0**.**0μg**/**kg**/**day**)*	dairy^b^	potatoes	meat^b^	freshwater fish^b^	pizza
Mean μg/kg/day	0.020878	0.006516	0.003474	0.01115	0.00151
n	199	101	94	62	61
% range	6.1-97.5%	1.1-81.6%	1.9-43.4%	1.8-86.8%	1.3-48.3%
** PCDD**/**Fs** (**0**.**002 ng**/**kg**/**day**)	dairy^b^	meat^b^	potatoes	cereal	mushrooms
Mean ng/kg/day	0.000469	0.000202	0.000139	0.000058	0.000155
n	196	128	39	39	39
% range	7.9-86.2%	3.1-60.0%	3.7-62.2%	2.6-19.8%	3.1-44.5%

^a^ Daily exposure totals (in μg/kg/day) calculated (in ppb), except PCDD/Fs, which are presented as ng/kg/day; reference dosages are presented in same units. Top sources were calculated by taking the top three food contributors to each individual’s exposure (per contaminant) and summarizing across the population the five foods appearing most commonly in the 3 highest ranks. The n refers to the number of participants for whom that food was among the top three contributors to intake for the given contaminant. The percentage range is the minimum to maximum contribution, across all individuals reporting that food item, for each of the main food items to total intake of the given contaminant.

^b^ Indicates a food subgroup. Dairy: all dairy and egg products; broccoli: broccoli, cauliflower, brussels sprouts; peaches: peaches, nectarines, plums; meat: beef, pork, lamb; poultry: chicken, turkey.

^c^ canned sweet potatoes.

**Figure 3:**
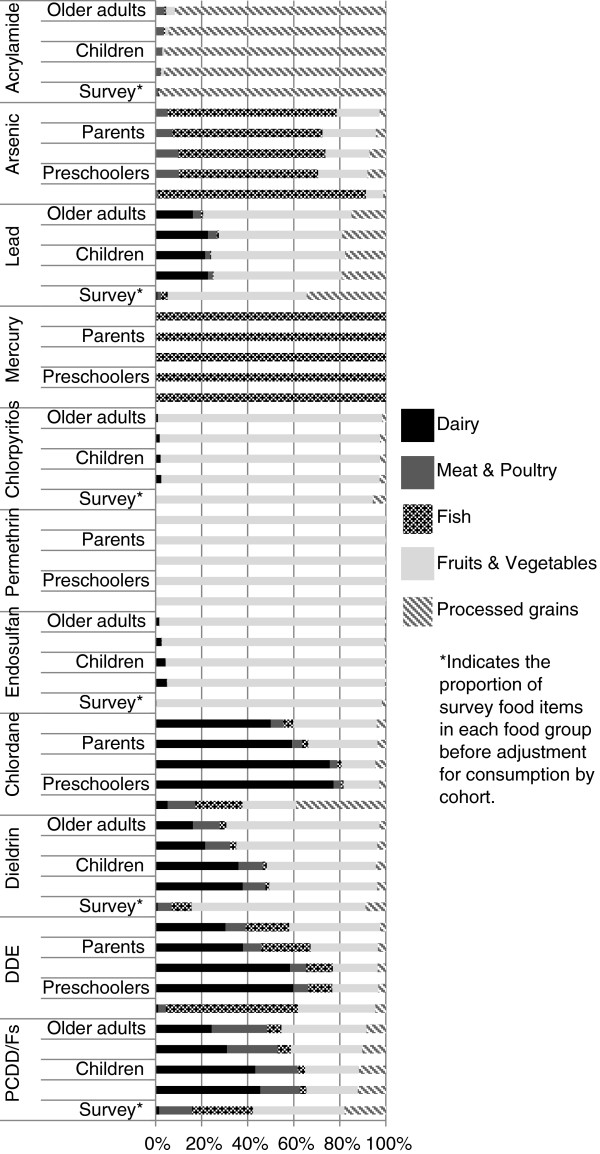
Percent contribution of food group to contaminant load by cohort.

We also grouped foods together to examine overall exposure to each contaminant by food group. Figure 3 shows the range in percentage contribution of intake for each contaminant by age group, which varied by food and consumption amounts. As expected, we found processed grains to be the primary source of acrylamide exposure as this compound is isolated from carbohydrate-based goods cooked at high temperatures. Fish was the primary route of exposure to arsenic and mercury. Fruits and vegetables were the primary source of dietary exposure to lead, chlorpyrifos, permethrin, endosulfan, and dieldrin. Dairy was the main source of chlordane, DDE, and PCDD/Fs exposure, although intake of PCDD/Fs was distributed across dairy, meat, and produce groups to varying degrees by age group with less exposure from dairy among adults due to lower dairy (and higher meat) consumption.

To examine the validity of reported levels of food intakes from the SUPERB survey, we compared the amounts of the top twenty most commonly consumed foods to the national averages by age group [37]. We found that across age groups, reported consumption of fruits, vegetables, and dairy in California tended to exceed national averages, poultry was comparable, and reported meat consumption tended to be lower than national averages though percentage differences between the two amounts were usually less than ten percent ( Additional file 4: Table S4).

## Discussion

### Contaminants that exceeded cancer and non-cancer benchmarks

For our study population, the means of the estimated intakes for the following dietary contaminants exceeded benchmark levels: were acrylamide, arsenic, lead, and among persistent organic pollutants, chlordane (children only), dieldrin, DDE, and PCDD/Fs. Children exceed the non-cancer and cancer benchmarks by a greater margin than adults for all compounds. This is especially of concern for children because all of these compounds are suspected endocrine disruptors and thus may impact normal development. Cancer risk ratios were exceeded by over a factor of 100 for arsenic and PCDD/Fs. Although we have emphasized compounds where the mean exceeded the benchmark, it is of public health consequence that more than 10% of the study population exceeded the non-cancer benchmarks for arsenic and methylmercury.

Health endpoints associated with contaminant exposure vary by compound. Acrylamide may induce neuromuscular defects [38]. Chronic arsenic exposure by ingestion has been related to various types of cancer [39]. Lead is known to damage the nervous and reproductive systems, especially in young children and at low levels with an approximate one point decrease in IQ with each 1 μg/dL increase in blood lead [40]. As for banned pesticides, chlordane exposure has been associated with cancers, neurotoxicity [41] and low birth weight [42]. Dieldrin has been linked to Parkinson's Disease and cancer [43]. DDE is genotoxic and an endocrine disruptor [44]. TCDD, the most toxic of the PCDD/F congeners, is an endocrine disruptor, is known to disrupt the developing immune, nervous, and reproductive systems, and has been shown to be teratogenic, mutagenic, carcinogenic, immunotoxic, and hepatotoxic in animal models [45,46].

Estimates of dietary exposures in SUPERB participants were similar to previously reported estimates of Dougherty et al. [13]. Both analyses found that cancer benchmarks were exceeded for arsenic, chlordane, dieldrin, DDT/DDE, and dioxin among children. Dougherty et al. reported that children were found to exceed lifetime benchmarks for intake of these pollutants by age 12 (a lifetime average daily dose based on childhood exposure alone was calculated to reach this conclusion).

Acrylamide was the only compound for which mean intakes in our study exceeded the noncancer benchmarks. By comparison, the only other study that included acrylamide was conducted in Japan and did not calculate acrylamide in terms of reference dosages [19]. Regarding the implications of high acrylamide exposure, the safety of dietary sources of acrylamide is currently under study by the FDA. A major goal of the draft FDA Action Plan on Acrylamide in Food, released in 2004, is to assess the dietary exposure of U.S. consumers to acrylamide by measuring acrylamide levels in various foods and estimating dietary exposure [47]. Findings on acrylamide exposure among children and adults reported herein contribute to this goal.

### Calculated intake compared to previous reports – dioxin

Previously reported intakes for dioxin were as follows: Jensen at al. quantified dioxin intake calculated as TEQ from fish and found that it ranged from 26–138 pg/person/day TEQ (2001). A study of Dutch children (n=207) ages 1–5 estimated cumulative TEQ (PCB-TEQ and dioxin-TEQ) intake from a food questionnaire at 6–7 pg/kg/day [48]. Our estimated levels were for only for PCDD/Fs TEQ (i.e., we did not include dioxin-like PCBs) and were therefore, as expected, considerably lower at 0.2-1.01 pg/person/day TEQ.

### Calculated intake compared to previous reports – arsenic, dieldrin, endosulfan, mercury, chlordane, permethrin, chlorpyrifos

In the analysis by Dougherty et al. [13], arsenic, dieldrin, endosulfan, mercury, chlordane, and permethrin intakes were found to be in a similar range as in SUPERB participants. Chlorpyrifos intake, however, was reported as 8.0 x10^-4^ mg/kg/day in Dougherty versus 7.1-7.5x10^-5^ in our study for children and 2.2x10^-5^ for adults. To seek an explanation for this discrepancy, we examined the difference in foods included in our analysis and found that the food sources we omitted were: rice, peas, oats, grapefruit, and cabbage. Though Dougherty et al. did not present their top contributors to chlorpyrifos because it was well below the noncancer reference dosage, we found apples, grapes, peaches, tomatoes, and peppers to be top contributors in our study population. Another possible explanation for the much lower chlorpyrifos intake levels in our study is the EPA regulation imposed in 2001 (after the Dougherty study was conducted) that banned chlorpyrifos for in-home use.

We found our arsenic intake estimates to fall below the non-cancer benchmarks but to greatly exceed the cancer benchmarks. However, this calculation of cancer risk is an overestimation because the food measurements of arsenic in the FDA Total Diet Study (the database used in this study) were made for total arsenic whereas EPA cancer benchmarks are set for inorganic arsenic. Inorganic arsenic, the more toxic form of arsenic, usually composes less than half of total arsenic. Previous studies of dietary exposures reconcile this discordance by citing a National Research Council analysis of the percentage of inorganic arsenic in total arsenic measurements made by the FDA Total Diet Study for 1991–1997 [49], which estimated inorganic arsenic intake to range from 0.066 to 0.34 μg/kg/day with an average of 0.14 μg/kg/day. Our estimated intake of total arsenic in SUPERB participants ranged from 0.14 (for older adults) to 1.22 (for preschool age children) μg/mg/kg. Considering that inorganic arsenic usually constitutes less than half of total arsenic, the estimates are actually within expected range for adult intake (estimated at 0.14-0.2 μg/kg/day). For children, our findings were more consistent with, or if anything on the low side in comparison with Yost and Tao et al., who found that children’s inorganic arsenic intake ranged from 1.6-6.2 μg/day with an average of 3.2 and that main sources were grain, fruits, rice, and milk (2004). Another study that analyzed inorganic arsenic in Total Diet Study foods found that inorganic arsenic was a higher percentage of total arsenic in rice and cereal grains compared to poultry and fish [23]. It is difficult to know whether the source attribution differences are due to regional variability in diet, as we found not only cereal, but also poultry, salmon, tuna, and mushrooms were top contributors to total arsenic intake. With a large coast and a sizable demographic of Asian ancestry in California, fish consumption does tend to be higher than elsewhere in the nation.

### Benchmarks

Though the reference dosages are meant to serve as benchmarks for safe exposure, there is debate in the scientific community about the validity of the term “safe exposure.” One weakness of benchmarks is that compounds are evaluated individually, whereas real life exposure scenarios involve multiple exposure routes and multiple compounds acting upon target organs and/or systems. Since exposures may operate synergistically, additively, or even antagonistically, a more comprehensive approach to establishing safe contaminant levels in food would consider the hundreds of chemicals humans are exposed to on a daily basis through a number of different routes and from different sources. Regarding benchmarks as indicators of safe exposure, there is evidence that disease occurs below the existing prescribed limits; thus allowances may need to be revised in accordance with estimates that take multiple exposures into account especially for highly sensitive groups (e.g. young children). For example, in a study of 7-year-olds (n=917) in which cognitive brain function was assessed in relation to prenatal methylmercury exposure, investigators reported that “early dysfunction is detectable at exposure levels currently considered safe” [50]. For lead, though there is no blood lead threshold for children by the EPA, the “action level” for clinical interventions was set at 10 μg/dL in 1991 by the CDC. Blood lead levels of concern have been progressively lowered since the 1960’s and despite observations of significant adverse effects occurring below a blood level of 10 μg/dL [51,52] no changes have been made in the lead standard.

### Top food contributors to contaminant exposure and dietary strategies to reduce contaminant intake – pesticides

The top contributors to pesticide intake among fruits and vegetables included tomatoes, peaches, apples, peppers, grapes, lettuce, broccoli, strawberries, spinach, pears, green beans, and celery. Six of the twelve top pesticide contributors from our study also appear on the Environmental Working Group’s Dirty Dozen for highest fruit and vegetable contributors of pesticide intake (peaches, apples, peppers, strawberries, spinach, and celery). While our calculations included a handful of common current use pesticides, those of the EWG included all of those tested by the FDA.

Dietary strategies to reduce exposure to food contaminants by necessity would vary by compound. Based on our calculations of food items that contribute most to intake in this sample, reducing exposure to pesticides is possible by substituting organic produce and milk for non-organic produce and milk. It may come as a surprise that milk, in addition to fruits and vegetables, was found to be a top contributor to intake of chlorpyrifos. This can be explained by the application of chlorpyrifos to grazing fields or feed given to dairy cattle, which is prohibited in organic milk production [53].

Consumers can potentially lower their exposure to current use pesticides by selecting types of conventionally grown produce with lower measured levels of pesticides. They can also purchase organically grown produce. Considering that some of the produce items high in pesticides are among the highest consumed fruits and vegetables in the U.S., it may be more effective to target agricultural production practices rather than consumer food choices to increase availability (and lower the cost) of organically grown tomatoes, peaches, apples, peppers, grapes, and strawberries, for example.

### Persistent organic pollutants & metals

Results from our study showed that milk was the leading dietary contributor of exposure to POPs for all age groups with animal foods and produce items making up the next four leading contributors. Fish was a major source of exposure resulting in arsenic, chlordane, dieldrin, dioxin, and DDT intake. Because POPs accumulate in animal fat, consuming a plant-based diet is one strategy to reduce exposure to POPs such as chlordane, DDE, and PCDD/Fs [54]. Another strategy to reduce POPs exposure is to decrease consumption of meat, dairy, and fish, or to select the lowest fat option. Fish is practically the sole source for methylmercury, but since levels are known to vary widely by fish species, consumers can avoid fish with high concentrations of mercury (shark and swordfish) in favor of fish and shellfish with low concentrations (e.g., catfish, canned salmon, and scallops) [55]. At the same time, consumers should recognize the health benefits of eating fish, and of particular importance for pregnant women and children, the well-established improvement in brain development from nutrients abundant in fish [56], but not in other foods.

While some produce items have measurable levels of lead and some POPs, organic produce consumption does not necessarily impact levels of metals or POPs. This is because accumulation of these compounds depends upon site-specific soil conditions that are not regulated by organic certification.

### Acrylamide

Reducing acrylamide in the diet can be accomplished by eliminating highly processed cereal, grain, and other carbohydrate products such as chips, cookies, French fries and crackers [57]. Lowering refined carbohydrate intake, particularly those high in saturated fats, trans fats, cholesterol, salt, and added sugar, can not only reduce acrylamide intake but also contribute to lower weight gain and improved glucose tolerance among the increasingly diabetic U.S. population [58].

### Study limitations

Limitations specific to the analysis herein relate to data collection in the SUPERB study and to complications inherent in estimating contaminant intake. These issues could have resulted in under- and over-estimations. Using the food frequency questionnaire allowed us to ask about foods known to be more heavily contaminated. However, to reduce participant burden, we asked about some foods as a group only, even though actual contaminant levels may vary by individual food item. For this reason, we recommend that future surveys ask about each food item individually to increase ease and accuracy of analysis. In our survey, if foods were grouped, the food consumed in greatest amounts (according to national averages) was selected to estimate the overall contaminant level. This might have skewed some results upward and others downward. For example, we asked about chips consumption in general, and while acrylamide levels are higher in potato chips (466.1 ppb) compared to tortilla chips (198.9 ppb), according to this procedure, we assigned the contaminant level for the most highly consumed item (tortilla chips have an average daily consumption of 5.6 grams compared to 4.0 grams for potato chips) which had lower contaminant levels and hence might have caused us to under-estimate exposure.

Exposures may have been overestimated if only a low percentage of items within a group exceeded the LOQ for particular contaminants (as was the case for chlorpyrifos in dairy). Benchmark hazard ratios for mercury levels may also have been overestimated because total mercury amounts were used while the benchmark level was for methylmercury only. However, according to our estimates, neither chlorpyrifos nor mercury intake exceeded benchmark values. Notably, the EPA’s cancer benchmark level for PCDD/Fs is three orders of magnitude higher than the WHO benchmark (which we applied to non-cancer endpoints), explaining why the cancer ratio of estimated risk to benchmark exceeds 100 while the non-cancer ratio of estimated hazard to benchmark is less than one. While the WHO benchmark is designated as appropriate for use in both cancer and non-cancer benchmarks, the EPA level was selected for consistency purposes in using EPA benchmarks, when available.

Other limitations relate to missing weight data and self-reported dietary data. Regarding missing data, imputations were made for a small percentage of adults (8.7%) and children (6.6%); we also calculated estimates based on non-imputed data and confirmed that the imputations did not bias our results. This study estimated contamination levels using published data from monitoring of food types and self-reported food consumption information rather than directly measuring those levels in the food consumed by study participants. Monitoring data sometimes indicate considerable variability across samples, though of course, each individual consumes many meals over the course of months or years, so that the use of averages is appropriate. Previous studies indicate that dietary surveys are a valuable tool for measuring food consumption, yet they share the same problems faced by all surveys: missing data and recall, reporting, and fatigue biases (i.e. long surveys). Of concern in self-reported dietary data is the potential for under-reporting of energy intake [59] which would lead to underestimation of toxic exposures. Previous research suggests that dietary survey respondents are inclined to over-report healthy foods and under-report unhealthy foods. A review by Carter and Whiting [60] found that about 80% of adult subjects under-reported what they ate and overall there was a tendency to under-report caloric intake by an average of 20-25%. A study on the validity of the FFQ found that foods most often over-reported were fruits and vegetables and that meat and dairy products were most often under-reported [61]. If these biases occurred for parents reporting on their children’s diet in the current study, we may have overestimated exposures to pesticides (chlorpyrifos, permethrin, and endosulfan) for which fruits and vegetables are the main source, and may have underestimated exposures to some of the persistent organic pollutants (e.g., chlordane, dieldrin, DDE, and PCDD/Fs), for which meat is a main source. On the other hand, results of a previous study validated dietary data collected from parents about their 3- to 5-year old children (similar to ages of SUPERB study children) by showing agreement between energy expenditure using doubly labeled water and diet history (similar to the food frequency questionnaire) [62].

## Conclusions

Despite challenges for data collection and analysis of food consumption and estimation of contaminant intake via food, the results we are reporting further our understanding of dietary contributions to toxic exposures. Findings can guide future examination of the multi-causal relationship between toxic exposure and health outcome, including food as one route of exposure. Based on dietary data we collected for different age groups, potential exposure to environmental toxins through the food consumption route is a real and significant concern particularly for children in their preschool and primary school years, with a high proportion of this age group estimated to exceed benchmark levels for a number of contaminants with known effects on health. Further studies are needed to understand the synergistic effects of exposure to multiple dietary toxins, the variability of cumulative dietary toxic exposure— particularly among young children—and the best approaches to limiting exposure to multiple compounds and from multiple routes.

## Abbreviations

TCDD: 2,3,7,8-Tetrachlorodibenzo-p-Dioxin; CPFs: Cancer potency factors; CSFII: Continuing Survey of Food Intakes by Individuals; DDE: Dichlorodiphenyldichloroethylene; EDCs: Endocrine disrupting contaminants; EWG: Environmental Working Group; FQPA: Food Quality Protection Act of 1996; PCDDs: Polychlorinated dibenzo-*p*-dioxins; PCDFs: Polychlorinated dibenzofurans; POPs: Persistent organic pollutants; SUPERB: Study of Use of Products and Exposure-Related Behavior; TDS: Total Diet Study; TEQ: Toxic equivalent; USDA: US Department of Agriculture; US EPA: US Environmental Protection Agency; US FDA: US Food and Drug Administration.

## Competing interests

The authors declare that they have no competing interests.

## Authors’ contributions

RV contributed to the development of dietary questionnaires, carried out the dietary and toxicological analysis, and prepared the manuscript. DB and DC designed the study and questionnaires, guided the analysis, and edited the manuscript. JF conducted programming and contributed to statistical analysis. BR designed the study and questionnaires, recruited the older subjects, and contributed to manuscript editing. IHP directed the project, led subject recruitment, designed the study and questionnaires, guided the analysis, and edited the manuscript. All authors read and approved the final manuscript.

## Authors' information

RV, is a professor of Public Health at Touro University and Principal Editor in the Office of Research at University of California, Davis. DB, DC, and IHP are professors in the Department of Public Health Sciences at University of California, Davis. JF was a programmer at University of California, Davis. BR is a professor of Epidemiology at the School of Public Health, University of California, Los Angeles.

## Supplementary Material

Additional file 1 **Table S1.** Variables Tested and Used for Subject Weight Imputation. Click here for file

Additional file 2 **Table S2.** Intake of food-based contaminants (mg/kg/day) among school-aged children, parents, and older adults (mg/kg/day). Click here for file

Additional file 3 **Table S3.** Top five food sources of contaminants for school-aged children, parents of young children, and older adults (mg/kg/day). Click here for file

Additional file 4 **Table S4.** Differences in Grams of Daily Food Intakes between Superb Data and National Data. Click here for file
