# Supplementary material for: Cancer and non-cancer health effects from food contaminant exposures for children and adults in California: a risk assessment
Source: Environ Health. 2012 Nov 9;11:83. doi: 10.1186/1476-069X-11-83 (PMC3551655; doi:10.1186/1476-069X-11-83)
Supplement: Additional file 1 — Table S1. Variables Tested and Used for Subject Weight Imputation. [file 1476-069X-11-83-S1.docx]

| Table S1. Variables Tested and Used for Subject Weight Imputation* | | | | |
| --- | --- | --- | --- | --- |
| Numeric Variables | Child Subjects | | Adult Subjects | |
| *Demographic Variables* | *Tested* | *Used* | *Tested* | *Used* |
| Has ≥ 1 allergies | Yes | Yes | No | No |
| Currently on a diet | No | No | Yes | Yes |
| Asian ethnicity | Yes | Yes | Yes | Yes |
| Has asthma | Yes | No | Yes | Yes |
| Born in the US | Yes | No | Yes | Yes |
| On a low-calorie diet | No | No | Yes | Yes |
| Has high cholesterol | No | No | Yes | Yes |
| Has diabetes | Yes | No | Yes | Yes |
| Has a college diploma | Yes | Yes | Yes | Yes |
| Has a high school diploma | Yes | Yes | Yes | Yes |
| Education level | Yes | Yes | Yes | Yes |
| has ≥1 food allergies | Yes | Yes | No | No |
| Height | Yes | Yes | No | No |
| Home owner | Yes | No | Yes | Yes |
| Has hypertension | No | No | Yes | Yes |
| Has internet | Yes | No | Yes | Yes |
| Latino/Hispanic race/ethnicity | Yes | No | Yes | Yes |
| Age | Yes | Yes | Yes | Yes |
| Is male | Yes | Yes | No | No |
| Took the survey in English | Yes | No | Yes | Yes |
| Total number of children in family | Yes | Yes | Yes | Yes |
| Vegetarian | Yes | Yes | No | No |
| North American or European origin | Yes | No | Yes | Yes |
| Caucasian race/ethnicity | Yes | Yes | Yes | Yes |
| *Food (in g per week)* | *Tested* | *Used* | *Tested* | *Used* |
| Apples | Yes | Yes | No | No |
| Applesauce | Yes | Yes | No | No |
| Green beans | Yes | No | Yes | Yes |
| Cantaloupe | Yes | Yes | No | No |
| Carrots | Yes | Yes | No | No |
| Celery | Yes | Yes | No | No |
| Cereal | Yes | Yes | Yes | Yes |
| Chicken | Yes | Yes | Yes | Yes |
| Chips | Yes | No | Yes | Yes |
| Cookies | Yes | No | Yes | Yes |
| Crackers | Yes | Yes | Yes | No |
| Cucumber | Yes | Yes | No | No |
| Donuts | Yes | Yes | No | No |
| Dried apricot | Yes | Yes | No | No |
| Dried fruit (other than apricot) | Yes | Yes | No | No |
| Fat grams per day | Yes | Yes | Yes | Yes |
| Fish | Yes | Yes | Yes | Yes |
| Fried potatoes | Yes | Yes | Yes | Yes |
| Freshwater fish | Yes | Yes | No | No |
| Gram crackers | Yes | Yes | Yes | Yes |
| Grams of food per week | Yes | No | Yes | Yes |
| Apple juice | Yes | Yes | Yes | No |
| Lettuce | Yes | Yes | No | No |
| Milk | Yes | No | Yes | Yes |
| Bell peppers | Yes | Yes | No | No |
| Pizza | Yes | Yes | Yes | Yes |
| Popcorn | Yes | Yes | Yes | Yes |
| Potatoes | Yes | Yes | Yes | Yes |
| Pretzels | Yes | Yes | Yes | No |
| Salmon | Yes | Yes | No | No |
| Seafood | Yes | Yes | No | No |
| Sweet potatoes | Yes | Yes | No | No |
| Tomatoes | Yes | Yes | No | No |
| Tortillas | Yes | No | Yes | Yes |
| Tuna | Yes | Yes | No | No |
| *1. Adults were analyzed in four cohorts (North and Central regions, by sex); children were analyzed in three cohorts (age 0-4, 5-10, 11-17 years). | | | | |
| 2. Variable is marked "used" if it was included in the imputation model of at least one of the respective Adult or Child cohorts. | | | | |
| 3. Variables were included if they were found to be correlated, or to have significantly different means, at the 20% level, but only if at least 5 True instances existed in the records which were not missing the subject weight. | | | | |
| 4. In this table, variables are listed in the Dietary domain only if they were calculated from reported food consumption. Questions about food allergies and dietary preferences are therefore listed here as Demographic information. | | | | |
