# Supplementary material for: Cancer and non-cancer health effects from food contaminant exposures for children and adults in California: a risk assessment
Source: Environ Health. 2012 Nov 9;11:83. doi: 10.1186/1476-069X-11-83 (PMC3551655; doi:10.1186/1476-069X-11-83)
Supplement: Additional file 2 — Table S2. Intake of food-based contaminants (mg/kg/day) among school-aged children, parents, and older adults (mg/kg/day). [file 1476-069X-11-83-S2.doc]

| Table S2 . Intake of food-based contaminants (mg/kg/day) among school-aged children, parents, and older adults (mg/kg/day) | | | | | | | | | | | | | | | |
| --- | --- | --- | --- | --- | --- | --- | --- | --- | --- | --- | --- | --- | --- | --- | --- |
|  |  |  |  |  | |  | |  | |  | |  |  |  |  |
| **School-aged Children (5-7) (n=157)*** | | | | | | | | | | | | | | |  |
|  | Descriptive exposure statistics . | | | | | | | | | | Benchmarks dosages . | | | |  |
| Toxin | N of exposed based on dietary data | Mean Daily mean per kg bodyweight^†^ | SD of daily mean (per kg bodywt) | | 10th percentile of daily mean (per kg bodywt) | | median of daily mean (per kg bodywt) | | 90th percentile of daily mean (per kg bodywt) | | Reference Dosage (RfD) (mg/kg/day) | % participants > RfD | Cancer Benchmark (CB) (mg/kg/day) | % participants > CB |  |
| **Acrylamide** | 157 | **9.10E-04** | 5.88E-04 | | 3.03E-04 | | 7.57E-04 | | 1.66E-03 | | 0.0002 | 95.54% | - | - |  |
| **Metals** |  |  |  | |  | |  | |  | |  |  | - |  |  |
| Arsenic | 157 | **1.87E-04** | 2.04E-04 | | 3.27E-05 | | 1.09E-04 | | 4.64E-04 | | 0.0003 | 18.47% | 0.0000125 | 100.00% |  |
| Lead | 157 | **1.15E-04** | 5.99E-05 | | 5.69E-05 | | 9.78E-05 | | 2.09E-04 | | 0.000 | 100.00% | - | - |  |
| Mercury | 111 | 3.04E-05 | 4.10E-05 | | 5.87E-06 | | 3.08E-05 | | 8.83E-05 | | 0.0001 | 6.31% | - | - |  |
| **Current use pesticides** | |  |  | |  | |  | |  | |  |  |  |  |  |
| Chlorpyrifos^a^ | 157 | 7.01E-05 | 5.99E-05 | | 1.42E-05 | | 5.95E-05 | | 1.26E-04 | | 0.003 | 0.00% | - | - |  |
| Chlorpyrifos^b^ | 74 | 6.34E-05 | 5.32E-05 | | 1.23E-05 | | 4.72E-05 | | 1.17E-04 | | 0.003 | 0.00% | - | - |  |
| Chlorpyrifos^c^ | 83 | 2.74E-05 | 3.63E-05 | | 6.82E-07 | | 1.89E-05 | | 8.04E-05 | | 0.003 | 0.00% | - | - |  |
| Permethrin^a^ | 157 | **1.22E-04** | 1.58E-04 | | 3.39E-06 | | 6.93E-05 | | 2.80E-04 | | 0.05 | 0.00% | 0.000056 | 56.05% |  |
| Permethrin^b^ | 74 | **1.23E-04** | 1.90E-04 | | 4.05E-06 | | 6.30E-05 | | 3.30E-04 | | 0.05 | 0.00% | 0.000056 | 51.35% |  |
| Permethrin^c^ | 79 | 4.79E-05 | 8.14E-05 | | 4.37E-08 | | 4.99E-06 | | 1.41E-04 | | 0.05 | 0.00% | 0.000056 | 34.18% |  |
| Endosulfan^a^ | 157 | 3.69E-05 | 2.75E-05 | | 8.25E-06 | | 3.21E-05 | | 7.39E-05 | | 0.006 | 0.00% | - | - |  |
| Endosulfan^b^ | 74 | 3.23E-05 | 2.36E-05 | | 7.43E-06 | | 2.59E-05 | | 7.27E-05 | | 0.006 | 0.00% | - | - |  |
| Endosulfan^c^ | 83 | 1.48E-05 | 1.92E-05 | | 3.99E-08 | | 7.45E-06 | | 3.99E-05 | | 0.006 | 0.00% | - | - |  |
| **Persistent organic pollutants** | | |  | |  | |  | |  | |  |  |  |  |  |
| Chlordane | 157 | **1.16E-05** | 6.31E-06 | | 4.01E-06 | | 1.11E-05 | | 2.01E-05 | | 0.0005 | 0.00% | 0.000001 | 99.36% |  |
| Dieldrin | 157 | **3.48E-06** | 2.61E-06 | | 1.40E-06 | | 2.87E-06 | | 6.00E-06 | | 0.00005 | 0.00% | 6.25E-08 | 100.00% |  |
| DDT | 157 | **2.74E-05** | 1.32E-05 | | 1.20E-05 | | 2.57E-05 | | 4.77E-05 | | 0.000 | 100.00% | 3.00E-06 | 100.00% |  |
| PCBs | 157 | 8.39E-10 | 3.56E-10 | | 4.29E-10 | | 8.02E-10 | | 1.41E-09 | | 5.00E-04 | 0.00% | 1.30E-07 | 0.00% |  |
|  |  |  |  | |  | |  | |  | |  |  |  |  |  |
| **Parents of Young Children (18-63) mean daily intake (n=446)*** | | | | | | | | | | | | | | |  |
|  | Descriptive exposure statistics . | | | | | | | | | | Benchmarks dosages . | | | |  |
| Toxin | N of exposed based on dietary data | Mean Daily mean per kg bodyweight^†^ | SD of daily mean (per kg bodywt) | | 10th percentile of daily mean (per kg bodywt) | | median of daily mean (per kg bodywt) | | 90th percentile of daily mean (per kg bodywt) | | Reference Dosage (RfD) (mg/kg/day) | % participants > RfD | Cancer Benchmark (CB) (mg/kg/day) | % participants > CB |  |
| **Acrylamide** | 446 | **2.12E-04** | 1.59E-04 | | 6.28E-05 | | 1.73E-04 | | 4.11E-04 | | 0.0002 | 43.50% | - | - |  |
| **Metals** |  |  |  | |  | |  | |  | |  |  | - |  |  |
| Arsenic | 446 | **7.74E-05** | 6.48E-05 | | 2.02E-05 | | 5.84E-05 | | 1.55E-04 | | 0.0003 | 1.12% | 0.0000125 | 96.19% |  |
| Lead | 446 | **2.44E-05** | 1.35E-05 | | 1.04E-05 | | 2.19E-05 | | 4.02E-05 | | 0.000 | 100.00% | - | - |  |
| Mercury | 446 | 1.45E-05 | 1.71E-05 | | 2.03E-06 | | 1.11E-05 | | 3.62E-05 | | 0.0001 | 0.74% | - | - |  |
| **Current use pesticides** | |  |  | |  | |  | |  | |  |  |  |  |  |
| Chlorpyrifos^a^ | 446 | 2.19E-05 | 1.69E-05 | | 4.52E-06 | | 1.79E-05 | | 4.69E-05 | | 0.003 | 0.00% | - | - |  |
| Chlorpyrifos^b^ | 201 | 1.89E-05 | 1.54E-05 | | 3.21E-06 | | 1.49E-05 | | 4.02E-05 | | 0.003 | 0.00% | - | - |  |
| Chlorpyrifos^c^ | 245 | 9.98E-06 | 1.25E-05 | | 1.81E-07 | | 5.42E-06 | | 2.61E-05 | | 0.003 | 0.00% | - | - |  |
| Permethrin^a^ | 446 | **1.09E-04** | 1.70E-04 | | 1.45E-05 | | 6.94E-05 | | 2.26E-04 | | 0.05 | 0.00% | 0.000056 | 60.54% |  |
| Permethrin^b^ | 201 | **9.53E-05** | 2.13E-04 | | 1.21E-05 | | 5.76E-05 | | 1.70E-04 | | 0.05 | 0.00% | 0.000056 | 51.24% |  |
| Permethrin^c^ | 241 | 4.72E-05 | 6.57E-05 | | 1.32E-08 | | 2.49E-05 | | 1.32E-04 | | 0.05 | 0.00% | 0.000056 | 28.22% |  |
| Endosulfan^a^ | 446 | 1.42E-05 | 9.93E-06 | | 3.60E-06 | | 1.18E-05 | | 2.86E-05 | | 0.006 | 0.00% | - | - |  |
| Endosulfan^b^ | 201 | 1.19E-05 | 8.70E-06 | | 2.86E-06 | | 1.02E-05 | | 2.29E-05 | | 0.006 | 0.00% | - | - |  |
| Endosulfan^c^ | 245 | 6.42E-06 | 7.23E-06 | | 2.29E-08 | | 4.50E-06 | | 1.65E-05 | | 0.006 | 0.00% | - | - |  |
| **Persistent organic pollutants** | | |  | |  | |  | |  | |  |  |  |  |  |
| Chlordane | 446 | **2.80E-06** | 1.60E-06 | | 1.11E-06 | | 2.47E-06 | | 5.01E-06 | | 0.0005 | 0.00% | 0.000001 | 92.15% |  |
| Dieldrin | 446 | **1.22E-06** | 9.60E-07 | | 4.39E-07 | | 9.42E-07 | | 2.39E-06 | | 0.00005 | 0.00% | 6.25E-08 | 100.00% |  |
| DDT | 446 | **8.75E-06** | 5.34E-06 | | 3.50E-06 | | 7.44E-06 | | 1.56E-05 | | 0.000 | 100.00% | 3.00E-06 | 93.50% |  |
| PCBs | 446 | 2.48E-10 | 1.17E-10 | | 1.19E-10 | | 2.24E-10 | | 3.97E-10 | | 5.00E-04 | 0.00% | 1.30E-07 | 0.00% |  |
|  |  |  |  | |  | |  | |  | |  |  |  |  |  |
| **Older Adults (>55) (n=149)*** | | | | | | | | | | | | | | |  |
|  | Descriptive exposure statistics . | | | | | | | | | | Benchmarks dosages . | | | |  |
| Toxin | N of exposed based on dietary data | Mean Daily mean per kg bodyweight^†^ | SD of daily mean (per kg bodywt) | | 10th percentile of daily mean (per kg bodywt) | | median of daily mean (per kg bodywt) | | 90th percentile of daily mean (per kg bodywt) | | Reference Dosage (RfD) (mg/kg/day) | % participants > RfD | Cancer Benchmark (CB) (mg/kg/day) | % participants > CB |  |
| **Acrylamide** | 149 | 1.56E-04 | 1.21E-04 | | 4.68E-05 | | 1.31E-04 | | 3.06E-04 | | 0.0002 | 21.48% | - | - |  |
| **Metals** |  |  |  | |  | |  | |  | |  |  | - |  |  |
| Arsenic | 149 | **8.41E-05** | 5.96E-05 | | 2.05E-05 | | 7.29E-05 | | 1.73E-04 | | 0.0003 | 0.67% | 0.0000125 | 96.64% |  |
| Lead | 149 | **2.19E-05** | 1.46E-05 | | 8.19E-06 | | 1.93E-05 | | 4.12E-05 | | 0.000 | 100.00% | - | - |  |
| Mercury | 149 | 1.50E-05 | 1.45E-05 | | 2.15E-06 | | 1.28E-05 | | 3.12E-05 | | 0.0001 | 0.00% | - | - |  |
| **Current use pesticides** | |  |  | |  | |  | |  | |  |  |  |  |  |
| Chlorpyrifos^a^ | 149 | 2.29E-05 | 1.92E-05 | | 5.11E-06 | | 1.73E-05 | | 5.05E-05 | | 0.003 | 0.00% | - | - |  |
| Chlorpyrifos^b^ | 98 | 2.16E-05 | 2.03E-05 | | 3.20E-06 | | 1.52E-05 | | 5.10E-05 | | 0.003 | 0.00% | - | - |  |
| Chlorpyrifos^c^ | 51 | 1.00E-05 | 1.29E-05 | | 1.90E-07 | | 6.74E-06 | | 2.25E-05 | | 0.003 | 0.00% | - | - |  |
| Permethrin^a^ | 149 | **9.29E-05** | 9.45E-05 | | 1.78E-05 | | 6.52E-05 | | 2.09E-04 | | 0.05 | 0.00% | 0.000056 | 55.03% |  |
| Permethrin^b^ | 98 | **7.78E-05** | 7.23E-05 | | 1.65E-05 | | 5.43E-05 | | 1.61E-04 | | 0.05 | 0.00% | 0.000056 | 47.96% |  |
| Permethrin^c^ | 51 | 3.32E-05 | 4.97E-05 | | 1.15E-08 | | 2.17E-05 | | 6.64E-05 | | 0.05 | 0.00% | 0.000056 | 17.65% |  |
| Endosulfan^a^ | 149 | 1.49E-05 | 1.03E-05 | | 4.11E-06 | | 1.20E-05 | | 2.92E-05 | | 0.006 | 0.00% | - | - |  |
| Endosulfan^b^ | 98 | 1.33E-05 | 1.01E-05 | | 3.44E-06 | | 1.09E-05 | | 2.55E-05 | | 0.006 | 0.00% | - | - |  |
| Endosulfan^c^ | 51 | 6.65E-06 | 7.07E-06 | | 3.73E-08 | | 5.21E-06 | | 1.50E-05 | | 0.006 | 0.00% | - | - |  |
| **Persistent organic pollutants** | | |  | |  | |  | |  | |  |  |  |  |  |
| Chlordane | 149 | **2.05E-06** | 1.01E-06 | | 7.71E-07 | | 1.92E-06 | | 3.43E-06 | | 0.0005 | 0.00% | 0.000001 | 84.56% |  |
| Dieldrin | 149 | **1.11E-06** | 9.61E-07 | | 3.27E-07 | | 8.11E-07 | | 2.37E-06 | | 0.00005 | 0.00% | 6.25E-08 | 100.00% |  |
| DDT | 149 | **6.87E-06** | 3.77E-06 | | 2.72E-06 | | 6.02E-06 | | 1.19E-05 | | 0.000 | 100.00% | 3.00E-06 | 88.59% |  |
| PCBs | 149 | 2.02E-10 | 8.79E-11 | | 9.64E-11 | | 1.96E-10 | | 3.13E-10 | | 5.00E-04 | 0.00% | 1.30E-07 | 0.00% |  |
| ^†^ Boldface values represent whole-population average estimated exposures that exceed the RfDs and/or CBs. | | | | | | | | | | | | | | |  |
| ^a^ All consumers of the food items related to this contaminant exposure. | | | | | | | | | | | |  |  |  |  |
| ^b^ The n and adjusted exposure level for those consumers who never purchase organic foods. | | | | | | | | | | | | |  |  |  |
| ^c^ The n and adjusted exposure level for consumers who did purchase three organic products some, most, or all of the time. | | | | | | | | | | | | |  |  |  |
