# Supplementary material for: Cancer and non-cancer health effects from food contaminant exposures for children and adults in California: a risk assessment
Source: Environ Health. 2012 Nov 9;11:83. doi: 10.1186/1476-069X-11-83 (PMC3551655; doi:10.1186/1476-069X-11-83)
Supplement: Additional file 3 — Table S3. Top five food sources of contaminants for school-aged children, parents of young children, and older adults (mg/kg/day). [file 1476-069X-11-83-S3.doc]

| Table S3 . Top five food sources of contaminants for school-aged children, parents of young children, and older adults (mg/kg/day) ^a^ | | | | | |
| --- | --- | --- | --- | --- | --- |
|  | **School-aged Children 5-7 yrs old (n=157)** | | | | |
| **Toxins (RfD)** | Highest | 2nd | 3rd | 4th | 5th |
| **Acrylamide (0.2)** | crackers | fried potatoes^b^ | cereal | chips | graham crackers |
| Mean mg/kg/day | 0.275643 | 0.257388 | 0.168540 | 0.202030 | 0.355345 |
| n | 90 | 84 | 73 | 72 | 70 |
| % range | 3.9-73.9% | 5.2-87.2% | 4.6-74.7% | 2.7-62.1% | 6.8-76.2% |
| **Metals** | Highest | 2nd | 3rd | 4th | 5^th^ |
| **Arsenic (0.3)** | poultry^a^ | salmon | cereal | tuna | mushrooms |
| Mean mg/kg/day | 0.019618 | 0.115467 | 0.012506 | 0.153371 | 0.057463 |
| n | 115 | 75 | 65 | 61 | 35 |
| % range | 1.5-82.6% | 4.9-89.7% | 2.7-66.5% | 3.9-88.4% | 3.4-72.3% |
| **Lead (0.0)*** | dairy^b^ | apple juice | grapes | carrots | cookies |
| Mean mg/kg/day | 0.026323 | 0.037854 | 0.016482 | 0.013837 | 0.012022 |
| n | 132 | 99 | 42 | 32 | 25 |
| % range | 3.8-76.9% | 6.7-75.3% | 5.2-24.6% | 7-31% | 3.7-25% |
| **Methylmercury (0.1)** | seafood^a^ | freshwater fish^b^ | tuna | n/a | n/a |
| Mean mg/kg/day | 0.024755 | 0.011228 | 0.027408 | n/a | n/a |
| n | 83 | 63 | 63 | n/a | n/a |
| % range | 6.5-100.0% | 1.1-100.0% | 2.1-100.0% | n/a | n/a |
| **Pesticides** | Highest | 2nd | 3rd | 4th | 5th |
| **Chlorpyrifos (3.0)** | apples | grapes | peaches | dairy^b^ | tomatoes |
| Mean mg/kg/day | 0.016029 | 0.025808 | 0.015287 | 0.00147 | 0.005323 |
| n | 133 | 133 | 109 | 32 | 22 |
| % range | 2.3-90.9% | 4.1-92.3% | 2.7-86.0% | 0.2-35.3% | 5.6-41.0% |
| **Permethrin (50.0)** | lettuce | spinach | broccoli^b^ | tomatoes | peaches |
| Mean mg/kg/day | 0.066259 | 0.128066 | 0.005857 | 0.015058 | 0.002814 |
| n | 90 | 76 | 65 | 62 | 50 |
| % range | 8-96.3% | 5.9-98.4% | 1-96.7% | 0.5-98.2% | 0.1-88.4% |
| **Endosulfan (6.0)** | apples | peaches | strawberries | tomatoes | pears |
| Mean mg/kg/day | 0.007423 | 0.007127 | 0.005403 | 0.00917 | 0.007574 |
| n | 97 | 80 | 70 | 60 | 32 |
| % range | 6.6-73.7% | 6.4-62.5% | 5.9-75.4% | 8.0-70.1% | 8.4-69.7% |
| **POPs** | Highest | 2nd | 3rd | 4th | 5th |
| **Chlordane (0.5)** | dairy^b^ | meat^b^ | cucumber | popcorn | potatoes |
| Mean mg/kg/day | 0.009475 | 0.006006 | 0.031685 | 0.010118 | 0.005442 |
| n | 154 | 74 | 46 | 61 | 43 |
| % range | 19.8-99.2% | 0.2-40.3% | 1.2-73.6% | 0.5-27.1% | 0.4-35.7% |
| **Dieldrin (0.05)** | dairy^b^ | meat^b^ | cucumber | cantaloupe | pizza |
| Mean mg/kg/day | 0.001219 | 0.000473 | 0.002132 | 0.000559 | 0.000166 |
| n | 149 | 91 | 69 | 61 | 20 |
| % range | 5-92.6% | 2.5-50.3% | 5.7-82.2% | 3.2-61.5% | 1.2-19.2% |
| **DDE (0.0)*** | dairy^b^ | meat^b^ | potatoes | freshwater fish^b^ | pizza |
| Mean mg/kg/day | 0.016211 | 0.002533 | 0.004072 | 0.009102 | 0.001301 |
| n | 153 | 89 | 73 | 50 | 33 |
| % range | 8.7-97.0% | 1.1-65.4% | 1.3-52.5% | 3.8-84.7% | 0.9-19.1% |
| **PCDD/Fs (0.002)** | dairy^b^ | meat^b^ | potatoes | poultry^a^ | cereal |
| Mean mg/kg/day | 0.000370 | 0.000162 | 0.000080 | 0.000065 | 0.000055 |
| n | 148 | 104 | 31 | 28 | 26 |
| % range | 7.7-84.2% | 2.9-61.5% | 2.9-25.4% | 3.0-17.1% | 3.0-24.1% |
|  | **Parents of Young Children 18-63 yrs old (n=447)** | | | | |
| **Toxins (RfD)** | Highest | 2nd | 3rd | 4th | 5th |
| **Acrylamide (0.2)** | fried potatoes^b^ | chips | cereal | crackers | cookies |
| Mean mg/kg/day | 0.06080 | 0.056279 | 0.047169 | 0.066287 | 0.042600 |
| n | 271 | 250 | 228 | 212 | 133 |
| % range | 1.1-85.2% | 1.6-68.8% | 2.3-93.9% | 3.9-85.0% | 3.8-72.4% |
| **Metals** | Highest | 2nd | 3rd | 4th | 5th |
| **Arsenic (0.3)** | tuna | salmon | poultry^a^ | mushrooms | cereal |
| Mean mg/kg/day | 0.038773 | 0.037196 | 0.005997 | 0.018472 | 0.00472 |
| n | 297 | 262 | 257 | 257 | 77 |
| % range | 5.3-94.0% | 1.7-93.9% | 1.3-80.0% | 0.8-95.7% | 2.8-42.1% |
| **Lead (0.0)*** | dairy^a^ | potatoes | apples | grapes | apple juice |
| Mean mg/kg/day | 0.005399 | 0.003547 | 0.003049 | 0.003752 | 0.005976 |
| n | 380 | 136 | 125 | 116 | 104 |
| % range | 5.2-70.8% | 5.0-71.5% | 4.1-64.4% | 3.8-50.0% | 4.0-67.9% |
| **Methylmercury (0.1)** | tuna | seafood^a^ | freshwater fish^b^ | salmon | n/a |
| Mean mg/kg/day | 0.006754 | 0.00999 | 0.004634 | 0.001055 | n/a |
| n | 318 | 281 | 258 | 217 | n/a |
| % range | 1.1-100.0% | 1.0-100.0% | 0.6-100.0% | 0.3-100.0% | n/a |
| **Pesticides** | Highest | 2nd | 3rd | 4th | 5th |
| **Chlorpyrifos (3.0)** | apples | grapes | peppers | tomatoes | peaches |
| Mean mg/kg/day | 0.00486 | 0.005449 | 0.00261 | 0.001458 | 0.005604 |
| n | 377 | 352 | 190 | 187 | 127 |
| % range | 1.8-96.7% | 2.6-93.0% | 1.0-91.5% | 1.5-62.7% | 3.3-88.7% |
| **Permethrin (50.0)** | lettuce | spinach | tomatoes | peppers | broccoli^b^ |
| Mean mg/kg/day | 0.033194 | 0.083694 | 0.005453 | 0.004755 | 0.001809 |
| n | 424 | 353 | 238 | 108 | 94 |
| % range | 2.5-98.1% | 3.7-99.7% | 0.4-76.3% | <0.1-77.3% | 0.6-33.5% |
| **Endosulfan (6.0)** | tomatoes | apples | peppers | beans | peaches |
| Mean mg/kg/day | 0.002933 | 0.002281 | 0.003294 | 0.000928 | 0.002724 |
| n | 312 | 275 | 231 | 100 | 88 |
| % range | 4.0-80.2% | 4.8-85.3% | 4.6-93.0% | 0.7-51.1% | 5.5-66.8% |
| **POPs** | Highest | 2nd | 3rd | 4th | 5th |
| **Chlordane (0.5)** | dairy^b^ | cucumber | meat^b^ | potatoes | spinach |
| Mean mg/kg/day | 0.001958 | 0.001041 | 0.000223 | 0.000241 | 0.000377 |
| n | 437 | 302 | 194 | 112 | 101 |
| % range | 5.6-96.0% | 1.1-93.2% | 1.1-40.9% | 0.7-48.8% | 0.8-53.9% |
| **Dieldrin (0.05)** | dairy^b^ | cucumber | meat^b^ | spinach | cantaloupe |
| Mean mg/kg/day | 0.000264 | 0.0006722 | 0.000159 | 0.000167 | 0.000213 |
| n | 396 | 332 | 290 | 88 | 57 |
| % range | 3.2-89.3% | 3.7-94.8% | 1.2-71.7% | 3.7-48.6% | 3.8-69.1% |
| **DDE (0.0)*** | dairy^b^ | potatoes | freshwater fish^b^ | meat^b^ | spinach |
| Mean mg/kg/day | 0.003445 | 0.001685 | 0.003759 | 0.000986 | 0.001679 |
| n | 419 | 233 | 206 | 192 | 142 |
| % range | 1.7-93.1% | 2.8-79.1% | 3.6-91.6% | 1.1-71.0% | 2.9-61.3% |
| **PCDD/Fs (0.002)** | dairy^b^ | meat^b^ | mushrooms | potatoes | poultry^a^ |
| Mean mg/kg/day | 0.000079 | 0.000054 | 0.000057 | 0.000032 | 0.000017 |
| n | 403 | 327 | 169 | 114 | 73 |
| % range | 6.3-70.7% | 4.2-61.2% | 3.9-71.1% | 2.4-59.9% | 2.0-39.0% |
|  | **Older Adults >55 yrs old (n=149)** | | | | |
| **Toxins (RfD)** | Highest | 2nd | 3rd | 4th | 5th |
| **Acrylamide (0.2)** | chips | fried potatoes^b^ | crackers | cereal | cookies |
| Mean mg/kg/day | 0.043596 | 0.045034 | 0.049502 | 0.038581 | 0.036293 |
| n | 78 | 73 | 70 | 68 | 49 |
| % range | 2.4-81.8% | 5.5-84.2% | 2.1-87.5% | 3.9-90.7% | 2.6-74.8% |
| **Metals** | Highest | 2nd | 3rd | 4th | 5th |
| **Arsenic (0.3)** | tuna | salmon | mushrooms | poultry^a^ | potatoes |
| Mean mg/kg/day | 0.040578 | 0.040991 | 0.016026 | 0.005771 | 0.004563 |
| n | 118 | 96 | 74 | 62 | 27 |
| % range | 5.9-95.4% | 2.2-89.1% | 1.8-88.1% | 1.3-47.8% | 1.3-34.9% |
| **Lead (0.0)*** | dairy^b^ | potatoes | sweet potatoes ^c^ | grapes | apples |
| Mean mg/kg/day | 0.003551 | 0.003446 | 0.007604 | 0.004636 | 0.002759 |
| n | 111 | 70 | 50 | 39 | 34 |
| % range | 5.5-62.5% | 6.6-49.4% | 6.9-64.6% | 6.5-56.3% | 4.1-40.3% |
| **Methylmercury (0.1)** | tuna | seafood^a^ | freshwater fish^b^ | salmon | n/a |
| Mean mg/kg/day | 0.00727 | 0.00808 | 0.003742 | 0.001285 |  |
| n | 122 | 118 | 71 | 69 | n/a |
| % range | 3-100.0% | 1.8-100.0% | 1.2-80.0% | 0.1-100.0% | n/a |
| **Pesticides** | Highest | 2nd | 3rd | 4th | 5th |
| **Chlorpyrifos (3.0)** | grapes | apples | tomatoes | peppers | peaches |
| Mean mg/kg/day | 0.006677 | 0.004247 | 0.001339 | 0.003296 | 0.008369 |
| n | 105 | 104 | 71 | 65 | 58 |
| % range | 3.5-90.7% | 2.2-90.2% | 1.0-74.4% | 3.5-73.9% | 11.6-86.9% |
| **Permethrin (50.0)** | lettuce | spinach | tomatoes | peppers | celery |
| Mean mg/kg/day | 0.03696 | 0.061324 | 0.005191 | 0.006226 | 0.00655 |
| n | 142 | 108 | 85 | 43 | 39 |
| % range | 2.4-97.3% | 4.7-94.9% | 1.6-95.4% | 0.3-62.5% | 0.8-43.8% |
| **Endosulfan (6.0)** | tomatoes | peppers | apples | peaches | beans |
| Mean mg/kg/day | 0.00295 | 0.004238 | 0.00213 | 0.003546 | 0.000638 |
| n | 108 | 73 | 68 | 51 | 33 |
| % range | 3.3-78.3% | 7.1-75.5% | 5.3-85.1% | 5.4-64.8% | 4.6-43.5% |
| **POPs** | Highest | 2nd | 3rd | 4th | 5th |
| **Chlordane (0.5)** | dairy^b^ | cucumber | meat^b^ | potatoes | salmon |
| Mean mg/kg/day | 0.001273 | 0.001124 | 0.000195 | 0.000241 | 0.000250 |
| n | 140 | 83 | 55 | 55 | 47 |
| % range | 4.9-97.6% | 5.0-87.7% | 1.1-37.7% | 1.2-68.9% | 1.6-77.4% |
| **Dieldrin (0.05)** | dairy^b^ | meat^b^ | cucumber | lettuce | spinach |
| Mean mg/kg/day | 0.000171 | 0.00014 | 0.000717 | 0.000066 | 0.00014 |
| n | 126 | 95 | 92 | 41 | 20 |
| % range | 3.3-83.9% | 2.7-67.9% | 7.1-93.6% | 1-31.3% | 4.7-39.7% |
| **DDE (0.0)*** | dairy^b^ | potatoes | meat^b^ | freshwater fish^b^ | spinach |
| Mean mg/kg/day | 0.002284 | 0.001807 | 0.000843 | 0.002882 | 0.00142 |
| n | 129 | 96 | 65 | 60 | 40 |
| % range | 6.9-84.1% | 4.6-82.4% | 3.4-60% | 2.1-74.7% | 4.1-79.2% |
| **PCDD/Fs (0.002)** | dairy^b^ | meat^b^ | potatoes | mushrooms | freshwater fish^b^ |
| Mean mg/kg/day | 0.000051 | 0.000047 | 0.000033 | 0.000048 | 0.000021 |
| n | 128 | 110 | 56 | 48 | 20 |
| % range | 8.3-60.0% | 6.4-75.3% | 5.1-40.1% | 7.3-65.0% | 4.7-20.7% |
| ^a^ Daily exposure totals (in mg/kg/day) calculated (in ppm). Numbers are presented as 10^3^ for all contaminants except PCDD/Fs, which are presented as 10^6^; reference dosages are also adjusted accordingly. Top sources were calculated by taking the top three food contributors to each individual’s exposure (per contaminant) and summarizing across the population the five foods appearing most commonly in the 3 highest ranks. The n refers to the number of participants for whom that food was among the top three contributors to intake of a given contaminant. The percentage refers to the range that the food item contributed to the total dietary intake of a given contaminant. | | | | | |
| ^b^ Indicates a food subgroup: | |  |  |  |  |
| dairy: all dairy and egg products | | |  |  |  |
| broccoli: broccoli, cauliflower, Brussels sprouts | | | |  |  |
| peaches: peaches, nectarines, plums | | |  |  |  |
| meat: beef, pork, lamb | |  |  |  |  |
| poultry: chicken, turkey | |  |  |  |  |
| ^c^ canned sweet potatoes | |  |  |  |  |
