# Supplementary material for: Cancer and non-cancer health effects from food contaminant exposures for children and adults in California: a risk assessment
Source: Environ Health. 2012 Nov 9;11:83. doi: 10.1186/1476-069X-11-83 (PMC3551655; doi:10.1186/1476-069X-11-83)
Supplement: Additional file 4 — Table S4. Differences in Grams of Daily Food Intakes between Superb Data and National Data. [file 1476-069X-11-83-S4.docx]

| Table S4 . Differences in Grams of Daily Food Intakes between Superb Data and National Data^†^ | | | | |
| --- | --- | --- | --- | --- |
| **Children (ages 2-6)** | | | | |
| Food | Superb Daily Averages (g) | National Daily Averages (g) | Difference (National-Superb) | Fractional Difference ((National-Superb)/National) |
| milk | 442.94 | 411.08 | -31.86 | -0.08 |
| apple juice | 110.67 | 54.89 | -55.78 | -1.02 |
| juice | 71.75 | 53.43 | -18.32 | -0.34 |
| applesauce | 49.70 | 11.15 | -38.55 | -3.46 |
| grapes | 44.68 | 5.02 | -39.66 | -7.90 |
| apples | 41.94 | 19.17 | -22.78 | -1.19 |
| potatoes | 30.78 | 4.12 | -26.67 | -6.48 |
| peaches | 27.03 | 6.57 | -20.47 | -3.12 |
| crackers | 24.78 | 3.73 | -21.06 | -5.65 |
| chicken | 24.60 | 23.80 | -0.80 | -0.03 |
| tomatoes | 23.60 | 2.33 | -21.27 | -9.13 |
| cereal | 22.70 | 18.72 | -3.99 | -0.21 |
| melon | 21.77 | 6.22 | -15.56 | -2.50 |
| broccoli | 20.12 | 3.96 | -16.17 | -4.09 |
| pears | 18.99 | 3.37 | -15.62 | -4.64 |
| cantaloupe | 18.76 | 3.95 | -14.81 | -3.75 |
| strawberries | 18.30 | 1.86 | -16.45 | -8.87 |
| carrots | 17.90 | 1.41 | -16.50 | -11.74 |
| beef | 17.42 | 35.68 | 18.26 | 0.51 |
| sweet potatoes | 14.36 | 1.21 | -13.16 | -10.92 |
| **Adults** | | | | |
| Food | Superb Daily Averages | National Daily Averages | Difference (National-Superb) | Fractional Difference ((National-Superb)/National) |
| milk | 337.43 | 220.11 | -117.32 | -0.53 |
| orange juice | 74.14 | 55.95 | -18.19 | -0.33 |
| apples | 52.43 | 12.74 | -39.69 | -3.12 |
| apple juice | 41.14 | 9.78 | -31.37 | -3.21 |
| potatoes | 40.00 | 13.06 | -26.95 | -2.06 |
| grapes | 36.14 | 2.60 | -33.54 | -12.90 |
| chicken | 30.71 | 41.78 | 11.07 | 0.26 |
| broccoli | 28.00 | 7.34 | -20.66 | -2.81 |
| tomatoes | 27.00 | 12.67 | -14.33 | -1.13 |
| beef | 26.71 | 64.37 | 37.65 | 0.58 |
| cereal | 25.43 | 12.79 | -12.64 | -0.99 |
| applesauce | 23.00 | 4.95 | -18.05 | -3.65 |
| pears | 18.57 | 2.05 | -16.52 | -8.07 |
| peaches | 17.29 | 5.42 | -11.87 | -2.19 |
| crackers | 16.43 | 3.80 | -12.63 | -3.33 |
| cantaloupe | 14.43 | 5.63 | -8.80 | -1.56 |
| cucumber | 14.29 | 3.32 | -10.97 | -3.30 |
| chips | 13.86 | 12.87 | -0.99 | -0.08 |
| beans | 13.29 | 7.08 | -6.21 | -0.88 |
| pizza | 12.57 | 21.46 | 8.89 | 0.41 |
|  |  |  |  |  |
| **Older Adults** | | | | |
| Food | Superb Daily Averages | National Daily Averages | Difference (National-Superb) | Fractional Difference ((National-Superb)/National) |
| milk | 242.71 | 226.95 | -15.77 | -0.07 |
| orange juice | 104.14 | 53.22 | -50.93 | -0.96 |
| potatoes | 54.29 | 19.93 | -34.36 | -1.72 |
| grapes | 44.71 | 4.02 | -40.70 | -10.13 |
| apples | 43.71 | 16.10 | -27.62 | -1.72 |
| apple juice | 39.14 | 7.96 | -31.18 | -3.92 |
| peaches | 39.00 | 9.84 | -29.16 | -2.96 |
| tomatoes | 33.00 | 18.03 | -14.97 | -0.83 |
| applesauce | 27.29 | 13.30 | -13.99 | -1.05 |
| beef | 25.43 | 55.00 | 29.57 | 0.54 |
| chicken | 25.43 | 29.04 | 3.61 | 0.12 |
| broccoli | 22.71 | 8.51 | -14.21 | -1.67 |
| cereal | 21.29 | 15.66 | -5.63 | -0.36 |
| pears | 19.71 | 5.60 | -14.12 | -2.52 |
| sweet potatoes | 19.57 | 3.63 | -15.95 | -4.40 |
| cucumber | 15.86 | 4.36 | -11.50 | -2.64 |
| cantaloupe | 14.71 | 8.31 | -6.40 | -0.77 |
| celery | 14.71 | 1.84 | -12.87 | -6.99 |
| lettuce | 14.29 | 15.85 | 1.56 | 0.10 |
| crackers | 14.00 | 4.05 | -9.95 | -2.46 |

^†^USEPA, 2003. CSFII Analysis of Food Intake Distributions. National Center for Environmental Assessment–Washington Office. Office of Research and Development
